# Phenotype of new daily persistent headache: subtypes and comparison to transformed chronic daily headache

**DOI:** 10.1186/s10194-023-01639-5

**Published:** 2023-08-16

**Authors:** Sanjay Cheema, Anker Stubberud, Khadija Rantell, Parashkev Nachev, Erling Tronvik, Manjit Matharu

**Affiliations:** 1 Headache and Facial Pain Group, University College London (UCL) Queen Square Institute of Neurology and The National Hospital for Neurology and Neurosurgery, Queen Square, London, UK; 2 The National Hospital for Neurology and Neurosurgery, Queen Square, London, UK; 3 High Dimensional Neurology Group, UCL Queen Square Institute of Neurology and National Hospital for Neurology and Neurosurgery, London, UK; 4 Department of Neuromedicine and Movement Sciences, NTNU Norwegian University of Science and Technology, Trondheim, Norway; 5 NorHEAD Norwegian Centre for Headache Research, NTNU Norwegian University of Science and Technology, Trondheim, Norway; 6 Education Unit, University College London (UCL) Queen Square Institute of Neurology, London, UK

**Keywords:** NDPH, Migraine, Chronic daily headache, Phenotype, Disease classification

## Abstract

**Background:**

It is unknown whether new daily persistent headache (NDPH) is a single disorder or heterogenous group of disorders, and whether it is a unique disorder from chronic migraine and chronic tension-type headache. We describe a large group of patients with primary NDPH, compare its phenotype to transformed chronic daily headache (T-CDH), and use cluster analysis to reveal potential sub-phenotypes in the NDPH group.

**Methods:**

We performed a case–control study using prospectively collected clinical data in patients with primary NDPH and T-CDH (encompassing chronic migraine and chronic tension-type headache). We used logistic regression with propensity score matching to compare demographics, phenotype, comorbidities, and treatment responses between NDPH and T-CDH. We used K-means cluster analysis with Gower distance to identify sub-clusters in the NDPH group based on a combination of demographics, phenotype, and comorbidities.

**Results:**

We identified 366 patients with NDPH and 696 with T-CDH who met inclusion criteria. Patients with NDPH were less likely to be female (62.6% vs. 73.3%, *p* < 0.001). Nausea, vomiting, photophobia, phonophobia, motion sensitivity, vertigo, and cranial autonomic symptoms were all significantly less frequent in NDPH than T-CDH (*p* value for all < 0.001). Acute treatments appeared less effective in NDPH than T-CDH, and medication overuse was less common (16% vs. 42%, *p* < 0.001). Response to most classes of oral preventive treatments was poor in both groups. The most effective treatment in NDPH was doselupin in 45.7% patients (95% CI 34.8–56.5%). Cluster analysis identified three subgroups of NDPH. Cluster 1 was older, had a high proportion of male patients, and less severe headaches. Cluster 2 was predominantly female, had severe headaches, and few associated symptoms. Cluster 3 was predominantly female with a high prevalence of migrainous symptoms and headache triggers.

**Conclusions:**

Whilst there is overlap in the phenotype of NDPH and T-CDH, the differences in migrainous, cranial autonomic symptoms, and vulnerability to medication overuse suggest that they are not the same disorder. NDPH may be fractionated into three sub-phenotypes, which require further investigation.

## Introduction

### Background

Chronic daily headache, meaning headache present on at least 15 days per month, is a major health problem affecting approximately 4% of the global population [1, 2]. New daily persistent headache (NDPH) is subtype of chronic daily headache, characterised by an acute onset daily continuous headache which persists for at least three months and has no identifiable secondary cause [3]. This is in contrast with most cases of chronic daily headache, which occur after a gradual transformation from an episodic headache disorder i.e., chronic migraine (CM) and chronic tension-type headache (CTTH) [3]. CM and CTTH may be grouped together as transformed chronic daily headache (T-CDH).

Despite the different tempo of onset, NDPH is often described in the literature as having a clinical phenotype resembling either CM or CTTH [4, 5]. The pathophysiology of NDPH is poorly understood but some authors have argued that the similarity in phenotype suggests that primary NDPH is simply de-novo CM or CTTH [6]. However, NDPH it is often highly refractory to treatments which are known to be effective in migraine [7–9], suggesting it may not have the same underlying biology.

It has been suggested that NDPH (even once secondary causes have been ruled out) may represent a group of disease processes rather than one homogenous disorder [10, 11]. If sub-phenotypes can be identified, this may potentially generate new pathophysiological hypotheses or help predict individual treatment response. Subtypes of primary NDPH have been proposed based on the precipitant for the headache, but these are based on anecdotal evidence [11]. NDPH may also be sub-classified according to whether it has a CM-like or CTTH-like phenotype, however it is not known whether either precipitant-based or phenotype-based subtypes of primary NDPH relate to either the pathophysiology of the headache or its response to treatment. Cluster analysis is an exploratory data analysis technique, which allows for data-driven identification of naturally occurring groups with similar characteristics. Due to the lack of biomarkers for headache disorders and their likely complex aetiology, we employed cluster analysis as a data driven, rather than intuitive, approach to explore for subgroups in the NDPH group [12].

### Objectives

Our primary objective was to compare the demographics, phenotype, comorbidities, and treatment responses of a large group of patients with NDPH to those with T-CDH; our secondary objective was to explore sub-phenotypes of primary NDPH using cluster analysis.

## Methods

### Participants

We performed a case–control study using prospectively collected clinical data in patients with primary NDPH and T-CDH. The population included all consecutive patients seen by a single neurologist (MSM) in a secondary and tertiary referral headache clinic at the National Hospital for Neurology and Neurosurgery, London, between 2007 and 2019 with a clinical diagnosis of NDPH, CM, or CTTH, for whom structured clinical records were available. In the NDPH group, patients were only included if they met International Classification of Headache Disorders 3^rd^ Edition (ICHD-3) criteria for NDPH [3], and if MRI brain had been performed and did not show evidence of a secondary cause. As per ICHD-3 criteria, patients with a prior history of infrequent episodic headache (defined by us as fewer than 5 headache days per month) were included, unless prior to the onset of NDPH they had reported an increasing headache frequency [3]. The T-CDH group was a combined group of patients with a clinical diagnosis of CM and CTTH, all of whom were experiencing an ongoing daily headache (headache on 28 days per month), which had persisted for at least three months. This definition differs from the definition of chronic daily headache commonly used in the literature (of headache on 15 or more days per month) in order for the population to be more comparable to those with NDPH [3]. Patients did not necessarily have to meet ICHD-3 criteria for CM or CTTH, as it is possible for patients to have an intermediate phenotype, which does not fully fit the criteria for either disorder [3]. For example, a patient with bilateral, non-pulsating pain, without aggravation by activity, but with nausea, photophobia, and phonophobia, is excluded from both migraine and tension-type headache criteria.

In both groups, patients were excluded if a secondary cause of the headache was identified or not thought to have been excluded; if there was a postural element to the headache; or if the diagnosis was later revised to a different primary headache syndrome. In both groups, patients with a strictly unilateral headache were excluded if a trial of indomethacin had not been performed to exclude hemicrania continua.

### Data collection

Data were extracted from semi-structured standardised clinical documents, which had been completed by a neurologist (MSM) prospectively as part of routine clinical care. All eligible patients had undergone a comprehensive clinical assessment including detailed systematic clinical phenotyping of the headache, recording of comorbidities, family history, and assessment of treatment responses. The majority of patients had completed the Headache Impact Test-6 (HIT-6) [13], Migraine Disability Assessment Scale (MIDAS) [14], and Hospital Anxiety and Depression Scale (HADS) questionnaires [15], These data used for the analysis were extracted in all patients using natural language processing techniques using methods described in a recent publication [16]. As part of data quality assurance, data which had been extracted using this automated method was compared in a random sample of 5% of patients in each of the NDPH and T-CDH groups to data extracted manually by one of the authors (SC). The precision (positive predictive value) and recall (sensitivity) of the automated data extraction compared to the manually extracted data were each approximately 90% and was similar in both groups. We employed strategies to ensure data completeness, however where missing data were unavoidable, patterns of missing data were investigated and none were found, and patients who were missing more than 10% of data were excluded from the final analysis. For most variables data was complete, but as no patterns of missing data were identified, where data remained missing it was excluded.

For the NDPH group, data on headache onset and precipitants (if any) were collected manually. An event was considered a precipitant if it occurred within seven days of onset of the headache and was considered to have precipitated the headache by both the patients and neurologist. For any pathology which may have caused a secondary headache in the acute stage, to be classified as a precipitant of primary NDPH the headache must have continued to have been daily and persistent for at least three months after the treatment or resolution of the original pathology, and there had to be no ongoing structural abnormality or neuropathy that explained the persistent pain.

We analysed response to acute treatments and oral preventive treatments for those treatments which had been used by at least 10% of patients in each group. Response to preventive medications was only assessed if the patient had completed at least a 3-month trial or the treatment was stopped sooner due to side effects. Treatment responses were graded based on the patients’ overall perception of improvement compared to baseline on a scale where 0 = no improvement and 100 = complete resolution of headaches, as follows: 1: no response or treatment failure (inability to tolerate due to side effects before reaching an effective dose), 2: mild response (1–29% improvement from baseline), 3: partial response (30–49% improvement from baseline), or 4: good response (50% or greater improvement from baseline).

### Statistical analysis

Descriptive data methods were used to summarise the demographic and clinical characteristics of the two groups. Continuous data were summarised using means with standard deviation (SD) or medians with interquartile range (IQR), depending on the distribution of data. Normality assumptions were assessed using visual inspection of histograms. Responses to treatment are presented as the proportion of patients who responded out of those who trialed each treatment.

For the comparison of clinical features between the NDPH and T-CDH groups, propensity score matching was used to account for the covariates of age, sex, and headache intensity [17]. Univariable and multivariable logistic regression were used to compare symptoms between the two groups [18]. The variables included were defined a priori, and were selected as they are the symptoms which are currently used for classification of primary headache disorders in ICHD-3 [3]. The variables assessed were headache laterality, throbbing pain quality, nausea, vomiting, photophobia, phonophobia, motion sensitivity, cranial autonomic symptoms, and aura symptoms.

Cluster analysis was performed including all patients with NDPH who had a complete dataset [19]. The variables included in the cluster analysis were selected a priori, and included demographics, phenotypic features, and comorbidities (see Table 3). Cluster analysis was performed using k-means clustering (k = 3) with Gower distance, which allows inclusion of both continuous numerical and categorical variables [20]. We also explored a variety of other linkage and distance methods, including, hierarchical clustering methods, but none gave a better separation of clusters. The number of clusters was determined by a high Calinksi-Harabasz pseudo-F-statistic [21]. To explore differences between the characteristics of the three clusters solution, we used one-way analysis of variance (ANOVA) to compare continuous variables between groups and Chi-squared test to compare categorical variables between groups. We also assessed whether the number of patients who responded to any preventive treatment or responded to any acute treatment (neither of which were included in the creation of the clusters) differed between the clusters.

Data cleaning and accuracy checking of automated data extraction were performed using Microsoft Excel. Descriptive data analysis was performed using SPSS Version 27, logistic regression and cluster analysis were performed using and Stata Version 11.2. *P* values shown are two-sided and unadjusted for multiple comparisons. Therefore, statistically significant results (*p* < 0.05) should be interpreted with caution.

### Standard protocol approvals, registrations, and patient consents

The initial process of organizing the data was performed in the clinical environment outside the study for the purpose of service improvement. Analysis of the anonymised data was performed under NRES approval by the London-West London & GTAC Research Ethics Committee (reference number 07/H0707/152) for the consentless analysis of irrevocably anonymized data.

## Results

### Participants

**Fig. 1:**
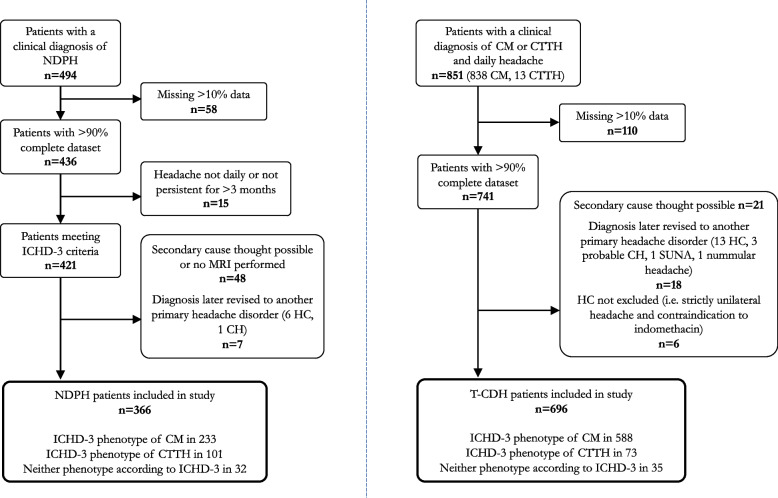
Flowchart of study participants and exclusions. CH, cluster headache; CM, chronic migraine; CTTH, chronic tension-type headache; HC, hemicrania continua; ICHD-3, international classification of headache disorders, 3^rd^ edition; MRI, magnetic resonance imaging; NDPH, new daily persistent headache; SUNA, short-lasting unilateral neuralgiform headache attacks

A total of 1345 patients with a daily headache were identified. Of these patients, 366 patients with NDPH and 696 with T-CDH met criteria for inclusion in the study (see Fig. 1). The vast majority (99%) of the patients in the T-CDH group had received a clinical diagnosis of CM, while only seven (1%) had a clinical diagnosis of CTTH. Age distribution and headache severity were similar in both groups but there was a lower female preponderance in NDPH (62.6%) than T-CDH (73.3%) (see Table 1).

**Table 1:** Demographics, headache severity, and comorbidities

	**NDPH** **(*n* = 366)**	**T-CDH** **(*n* = 696)**
Age (mean ± SD)	37.9 ± 14.4	40.3 ± 15.2
Sex, females (frequency, percentage)	229 (62.6%)	510 (73.3%)
Headache frequency, days per month (mean ± SD)	28 ± 0	28 ± 0
Headache intensity, 0–10 scale (mean ± SD)	7.65 ± 1.63	8.15 ± 1.32
Medication overuse	57 (15.6%)	293 (42.1%)
Clinical diagnosis of depression	74 (20.2%)	166 (23.8%)
Clinical diagnosis of anxiety	58 (15.8%)	122 (17.5%)
Clinical diagnosis of joint hypermobility syndrome	41 (11.2%)	129 (18.5%)
HIT 6	*n* = 278	*n* = 517
Median score (range)	67 (36–78)	67 (36–78)
Proportion in severe impact range (score ≥ 60)	242 (87.1%)	471 (91.1%)
MIDAS	*N* = 273	*N* = 493
Median score (range)	90 (0–270)	84 (0–270)
Proportion in severe impact range (score ≥ 21)	238 (87.2%)	430 (87.2%)
HADS-A	*N* = 282	*N* = 518
Median score (range)	8 (0–21)	9 (0–21)
Proportion in abnormal range (score ≥ 11)	100 (35.5%)	204 (39.4%)
HADS-D	*N* = 282	*N* = 520
Median score (range)	8 (0–21)	8 (0–21)
Proportion in abnormal range (score ≥ 11)	110 (39.0%)	167 (32.1%)

*HADS-A* Anxiety subset of Hospital Anxiety and Depression Scale; *HADS-D* Depression subset of Hospital Anxiety and Depression Scale, *HIT-6*, six-item Headache Impact Test, *MIDAS* Migraine Disability Assessment Test

Overall, 140/366 (38.3%) of the patients with NDPH had a precipitant. The most common precipitant was an influenza-like viral illness, which occurred in 56 patients (40% of those with a precipitant). A full list of headache precipitants is shown in Table 2. Twenty-three patients (6.3%) had a thunderclap headache at onset.

**Table 2:** Precipitants for new daily persistent headache

Precipitant	Number of patients and proportion of those with a precipitant
Systemic / extracranial infection (flu-like illness × 28, upper respiratory tract infection × 9, gastrointestinal infection × 8, urinary tract infection × 2, respiratory infection × 2, Lyme disease × 2, labyrinthitis × 1, malaria × 1, mycoplasma × 1, otitis media × 1, shingles × 1)	56 (40%)
Stressful life event	14 (10%)
Cranial infection (viral meningitis × 7, meningoencephalitis × 2, sinusitis × 2)	11 (7.9%)
Cranial surgery/procedure (dental surgery × 6, excision of acoustic neuroma × 1, laser eye surgery × 1, excision of cervical lesion × 1, exploration of frontal region × 1, temporomandibular joint surgery × 1)	11 (7.9%)
Drug (combination of cocaine and amphetamines × 1, venlafaxine × 1, sildenafil × 1, flu vaccine × 1, GCSF × 1, isotretinoin × 1, MDMA × 1, flecainide × 1, withdrawal from venlafaxine × 1, withdrawal from multiple psychiatric drugs [methylphenidate, pregabalin, quetiapine, and diazepam] × 1)	10 (7.1%)
Cranial vascular (carotid dissection × 2, venous sinus thrombosis × 2, vertebral artery dissection × 1, subarachnoid haemorrhage 1)	6 (4.3%)
Extracranial injury (lower back injury × 3, neck injury × 1, shoulder injury × 1, left arm injury × 1)	6 (4.3%)
Extracranial surgery/procedure (coronary angiogram 1, lumbar spine surgery 1, sterilisation surgery 1, thyroidectomy 1, wrist ganglionectomy 1)	5 (3.6%)
Other (syncope 3, exertion 3, insect bites 2, choking episode 1, corneal abrasion 1, during blood test 1, exhaustion 1, flight 1, high altitude cerebral oedema 1, inflammatory 3^rd^ cranial nerve palsy 1, pre-eclampsia 1, semicircular canal dehiscence 1)	21 (15%)

*GCSF* Granulocyte colony-stimulating factor, *MDMA* 3,4-Methylenedioxymethamphetamine

### Headache phenotype

**Fig. 2:**
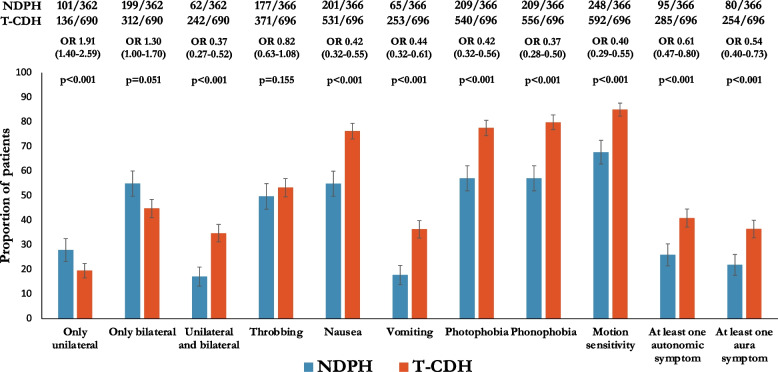
Headache characteristics in NDPH and T-CDH. OR, odds ratio; NDPH, new daily persistent headache; T-CDH, transformed chronic daily headache (encompassing chronic migraine and chronic tension-type headache). Error bars indicate 95% confidence intervals

In a significantly lower proportion of patients in the NDPH group, the headache was associated with each of the following characteristics: nausea, vomiting, photophobia, phonophobia, motion sensitivity, cranial autonomic symptoms, and aura symptoms (see Fig. 2). Patients with NDPH were more likely to have a strictly unilateral headache, whereas those with T-CDH were more likely to have both unilateral and bilateral headaches. The proportion of patients who had a throbbing pain quality was similar in each group (see Fig. 2).

Based on the headache phenotype alone, 233 (64%) of patients in the NDPH group would have met ICHD-3 criteria for CM, 101 (28%) criteria for CTTH, and 32 (9%) neither criteria. In the T-CDH group, 588 (84%) met criteria for CM, 73 (10%) for CTTH, and 35 (4%) neither criteria.

### Comorbidities

The three most common comorbidities in each groups were clinically-diagnosed depression, anxiety, and joint hypermobility disorders (see Table 1). In both groups, most patients were in the highly disabled range for both HIT-6 (score ≥ 60) and MIDAS (score ≥ 21) and there was a similar proportion of anxiety and depression measured using HADS in each group (see Table 1).

The presence of medication overuse (diagnosed according to ICHD-3 criteria depending on the type of acute medication which was overused) was far less common in NDPH compared to T-CDH (15.6% compared to 42.1%).

### Acute treatments

**Fig. 3:**
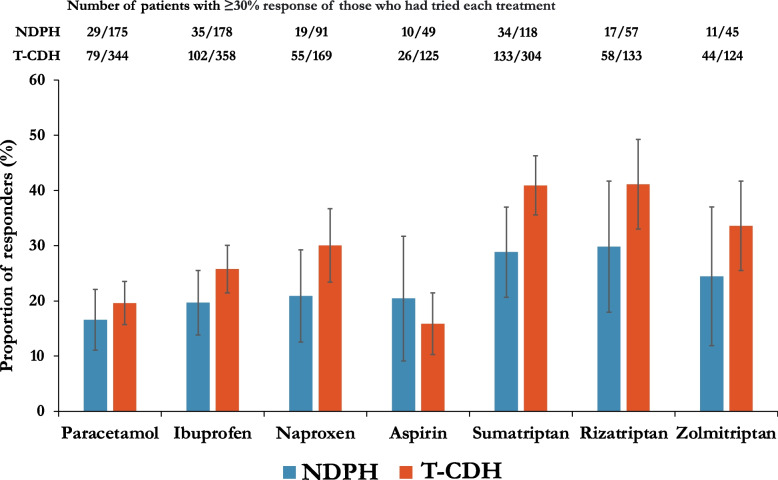
Acute treatment responses, NDPH, new daily persistent headache; T-CDH, transformed chronic daily headache (encompassing chronic migraine and chronic tension-type headache). Error bars indicate 95% confidence intervals

The same acute treatments had been used by 10% or more patients in each group and were therefore included in the analysis (see Fig. 3). There was a trend for both NSAIDs and triptans to be less effective in NDPH than T-CDH.

### Preventive treatments

**Fig. 4:**
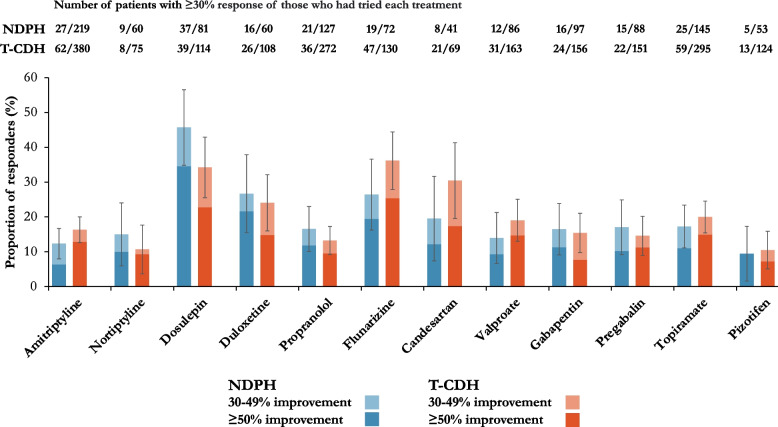
Preventive treatment responses. NDPH, new daily persistent headache; T-CDH, transformed chronic daily headache (encompassing chronic migraine and chronic tension-type headache). Error bars indicate 95% confidence intervals

The same preventive treatments had been used by 10% or more patients in each group and were therefore included in the analysis (see Fig. 4). The response to most preventive treatments in both groups was poor, with less than 20% of patients reporting even a 30% improvement with many of the treatments. The most effective treatment in the NDPH group was doselupin with 45.6% (95% CI 35–56%) experiencing at least a 30% improvement.

### Cluster analysis

**Fig. 5:**
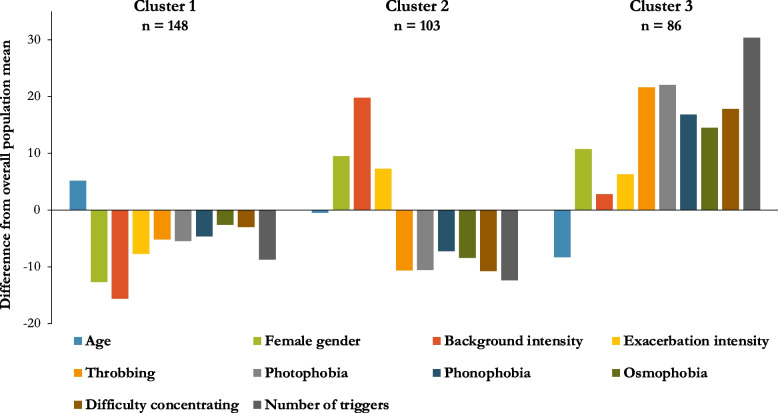
Discriminatory variables in cluster analysis of NDPH, Only variables which were statistically significant different after correction for multiple comparisons between the three groups are shown. See Table 3 for details of all included variables.

Cluster analysis was performed in the 337 of the 366 patients who did not have any missing data. A three-cluster solution was determined, including 29 variables (see Table 3). Cluster 1 was older, had a high proportion of male patients, and had less severe headaches. Cluster 2 was predominantly female, had severe headaches, and a low prevalence of migrainous symptoms. Cluster 3 was also predominantly female but had a high prevalence of migrainous symptoms and high number of headache triggers (see Fig. 5).

**Table 3:** Demographic and clinical characteristics of each cluster

	**Cluster 1** ***n* = 148**	**Cluster 2** ***n* = 103**	**Cluster 3** ***n* = 86**	***P* value**^**#**^
Age (years), mean ± SD	41.4 ± 13.3	36.7 ± 15.2	30.2 ± 11.0	< 0.001*
Female sex	72 (48.7%)	73 (70.9%)	62 (72.1%)	< 0.001*
Background intensity (0–10 VRS), mean ± SD	3.2 ± 1.0	6.8 ± 1.5	5.1 ± 1.7	< 0.001*
Exacerbation intensity (0–10 VRS), mean ± SD	6.9 ± 1.7	8.4 ± 1.1	8.3 ± 1.2	< 0.001*
Only bilateral	85 (57.4%)	61 (59.2%)	45 (52.3%)	0.616
Only unilateral	40 (27.0%)	32 (31.1%)	19 (22.1%)	0.384
Bilateral and unilateral	23 (15.5%)	10 (9.7%)	22 (25.6%)	0.013
V1 pain	118 (79.7%)	73 (70.9%)	77 (89.5%)	0.007
V2 pain	16 (10.8%)	15 (14.6%)	15 (17.4%)	0.344
V3 pain	99 (66.9%)	61 (59.2%)	63 (73.3%)	0.123
Occipital pain	102 (57.4%)	67 (52.4%)	54 (46.5%)	0.268
Neck pain	20 (13.5%)	17 (16.5%)	19 (22.1%)	0.236
Throbbing quality	67 (45.3%)	41 (39.8%)	62 (72.1%)	< 0.001*
Nausea	75 (50.7%)	58 (56.3%)	53 (61.6%)	0.258
Vomiting	14 (9.5%)	24 (23.3%)	21 (24.4%)	0.003
Photophobia	78 (52.7%)	49 (47.6%)	69 (80.2%)	< 0.001*
Phonophobia	80 (54.1%)	53 (51.5%)	65 (75.6%)	0.001*
Osmophobia	23 (15.5%)	10 (9.7%)	28 (32.6%)	< 0.001*
Motion sensitivity	98 (66.2%)	67 (65.1%)	72 (83.7%)	0.007
Difficulty concentrating	69 (46.6%)	40 (38.8%)	58 (67.4%)	< 0.001*
Vertigo	21 (14.2%)	15 (14.6%)	27 (31.4%)	0.002
Cranial autonomic symptoms	63 (42.6%)	49 (47.6%)	43 (46.0%)	0.507
Aura	26 (17.6%)	24 (23.3%)	23 (26.7%)	0.231
Number of triggers, mean ± SD	2.2 ± 1.7	1.8 ± 1.4	6.5 ± 1.9	< 0.001*
Medication overuse	25 (16.9%)	18 (17.5%)	9 (10.5%)	0.333
Hypermobility	9 (6.1%)	13 (12.6%)	18 (20.9%)	0.003
Depression	23 (15.5%)	23 (22.3%)	22 (25.6%)	0.147
Anxiety	19 (12.8%)	18 (17.5%)	20 (23.3%)	0.120
Family history of migraine	63 (42.6%)	42 (40.8%)	49 (57.0%)	0.050

^**#**^*P* values represent one-way analysis of variance (ANOVA) to compare continuous variables between groups and Chi-squared test to compare categorical variables between groups

^*^Indicates those *p* values which are below the critical *P* value adjusted from 0.05 to 0.00172 after Bonferroni correction with 29 comparison

The three clusters had a similar proportion of patients who responded to any preventive treatment. A greater proportion of patients who responded to any acute treatment was noted in cluster 3, compared to clusters 1 and 2 (48% vs. 30% and 24% respectively) (X^2^ = 12.4 (df = 2, *N* = 337), *p* = 0.002).

## Discussion

The characteristics of this large group of patients with NDPH are similar to those found in a recent meta-analysis of primary NDPH, and therefore it is likely to be a representative sample [4]. We confirm that NDPH is precipitated in approximately 40% of cases, most commonly following a flu-like viral illness, but also from a variety of other newly described events (see Table 2). These precipitants do not have an obvious common theme, although many are causes of acute headache, which suggests that NDPH may be a result of a triggered central or peripheral sensitisation. Some of the described precipitants may be coincidental, but we attempted to minimise this by only including those which occurred within seven days of NDPH-onset and where both the patients and clinician considered the event to have triggered the headache. Similarly to other studies, we found that approximately 2/3 of patients with NDPH had a CM phenotype and 1/3 a CTTH phenotype [4].

To determine whether there were differences between NDPH and other primary chronic daily headache disorders we elected to combine patients with CM and CTTH for comparison, as NDPH can have either phenotype, and in both the NDPH and T-CDH groups we saw a spectrum in the number of migraine symptoms rather than a clear divide between the migraine and tension-type phenotypes. We found that in comparison to T-CDH, despite a similar headache severity in the two groups, patients with NDPH are less likely to have migrainous symptoms, cranial autonomic symptoms, and aura symptoms. Other smaller studies have also recently sought to compare the phenotype of NDPH to other chronic daily headache disorders. A paediatric study comparing NDPH to T-CDH found that, other than less photophobia in the NDPH group, the two groups had a similar phenotype [22]. Like NDPH, the phenotype of chronic post-traumatic headache also commonly resembles migraine in approximately 2/3 of cases and tension-type headache in approximately 1/3, and its differentiation from these diagnoses is under debate [23, 24]. A study has compared 50 adolescents each with NDPH, CM, and chronic persistent post-traumatic headache, and did not find significant differences [25]. These two studies suggest that our findings may not apply to child and adolescent patients with chronic daily headache. The only other adult study comparing NDPH-CM to transformed CM did find several differences between the groups. Even though only those patients with NDPH who had a migraine phenotype were included in that study, the NDPH group had fewer migraine symptoms, less osmophobia, and less nausea and vomiting, in keeping with our findings [26]. If the underlying biology was similar, it is unclear why a sudden onset chronic daily headache should have a different phenotype to one with a transformed onset. This suggests that at least in adults, some patients with NDPH are likely to have a different pathophysiology.

Joint hypermobility disorders have previously been linked to an increased prevalence of headache, particularly NDPH [27, 28]. In our study, the high proportion of patients in both groups with a diagnosis of a hypermobility disorder is likely affected by local factors, being within the same hospital trust as a national referral joint hypermobility service. In our population, a diagnosis of a hypermobility disorder was unexpectedly more common in the T-CDH group than the NDPH group, which could not have been explained by local referral factors. This suggests that joint hypermobility is likely a predisposing factor for developing chronic headache, rather than NDPH specifically. Both NDPH and T-CDH had high levels of headache-related disability, anxiety, and depression, which were similar to previously published scores in CM [29, 30]. Diagnoses of depression and anxiety were both slightly less common in NDPH than T-CDH. Therefore, unlike a recent study [31], our results do not support the notion that NDPH has any more of a psychological basis than other forms of CDH.

Our evidence for treatment response is based on the results of a prospective, observational, non-randomised, uncontrolled study; and treatment response was assessed by patients’ global perception of improvement rather than headache diaries. We acknowledge the inability to control for bias inherent to such design and have implemented measures to minimise this. Despite this, in the absence of any controlled trials in primary NDPH, this study represents the largest dataset for treatment of NDPH in the literature. We have confirmed NDPH to be a highly treatment-refractory disorder, at least in the population referred to specialist headache clinics. Our results suggest that acute treatments may be less effective in NDPH than T-CDH. This may the reason that medication overuse was much less common in NDPH than T-CDH, a fact that has also been demonstrated in a paediatric study [22]. We expected that patients with NDPH may respond less well to preventive treatments than those with T-CDH, as previous studies have shown that even infusion treatments such as dihydroergotamine [32], and invasive surgical treatment with occipital nerve stimulation appear less effective in NDPH than CM [33]. However, we found that responses in both groups were poor. Patients with a daily continuous headache have not been included in trials for many treatments for migraine, and the presence of a daily headache has been identified as a negative predictor of response to treatments including Candesartan and Erenumab [34–36], suggesting that these patients (whether NDPH or T-CDH) may require a different treatment strategy. As this study was conducted in a predominantly tertiary referral clinic, we will likely have underestimated the efficacy of more widely used oral preventive medications (e.g., amitriptyline, topiramate, and propranolol) as patients will often have tried and failed treatment with these medications before being referred to our clinic. The superior efficacy of doselupin compared with the other tricyclic antidepressants amitriptyline and nortriptyline is likely due to these reasons as well as its lower incidence of side effects, and shorter time to titrate to an effective dose. Unfortunately, doselupin is not available in many countries and in the UK doselupin is difficult to prescribe within the National Health Service as depression guidelines recommend against its use due to risk of toxicity in overdose [37].

Using cluster analysis, we found three groups which differed on the basis of age, sex, headache intensity, associated features, and triggerability (see Table 3 and Fig. 5). Cluster 3, affecting young females, is highly suggestive of a migrainous biology. This may account for the degree in overlap of the phenotype of NDPH and T-CDH. The mechanisms of cluster 1 (less severe and affecting older male patients) and cluster 2 (very severe but with few associated symptoms) are unclear. To the best of our knowledge, this type of analysis has not previously been performed in NDPH. One published study has attempted cluster analysis in chronic migraine, however the results appeared to show three clusters on a spectrum, in contrast to the clusters we identified within the NDPH which varied according to multiple dimensions [38]. Our findings are exploratory and require further testing. All methods of cluster analysis share the limitation that potential clusters may be found which do not exist in the real world, and we need to determine the biological basis of these groups and whether similar clusters are reproducible in other cohorts of NDPH.

In conclusion, whilst there is overlap in the phenotype of NDPH and T-CDH; NDPH is a more featureless disorder, less likely to have both migrainous and cranial autonomic features, despite similarly high headache severity and disability levels. We confirm the suspicions of other authors that primary NDPH is often refractory to treatment. These results in conjunction suggest that NDPH may have a different pathophysiology to CM and CTTH, and that NDPH may require a different treatment paradigm. We propose that for now, NDPH should be considered a distinct disorder from CM and CTTH. In fact, NDPH may be comprised of three different phenotypes, which require further investigation.

## Data Availability

Anonymised data used in this study are available upon reasonable request to the corresponding author by any qualified investigator.
